# Sirolimus inhibits key events of restenosis in vitro/ex vivo: evaluation of the clinical relevance of the data by SI/MPL- and SI/DES-ratio's

**DOI:** 10.1186/1471-2261-7-15

**Published:** 2007-05-11

**Authors:** Rainer Voisard, Svenja Zellmann, Fabian Müller, Felicitas Fahlisch, Lutz von Müller, Regine Baur, Jürgen Braun, Jürgen Gschwendt, Margaratis Kountides, Vinzenz Hombach, Joachim Kamenz

**Affiliations:** 1 Department of Internal Medicine II – Cardiology, University of Ulm, Robert-Koch-Straße 8, D-89081 Ulm, Germany; 2 Department of Virology, Institute of Mikrobiology and Immunology, University of Ulm, Robert-Koch-Straße 8, D-89081 Ulm, Germany; 3 Department of Urology, Klinik Biberach, Ziegelhausstraße 50, D-88400 Biberach, Germany; 4 Department of Urology, University of Ulm, Prittwitzstrasse 43, D-89075 Ulm, Germany; 5 Department of Urology, Klinik Heidenheim, Schlosshaustraße 100, D-89522 Heidenheim, Germany

## Abstract

**Background:**

Sirolimus (SRL, Rapamycin) has been used successfully to inhibit restenosis both in drug eluting stents (DES) and after systemic application. The current study reports on the effects of SRL in various human in vitro/ex vivo models and evaluates the theoretical clinical relevance of the data by SI/MPL- and SI/DES-ratio's.

**Methods:**

Definition of the SI/MPL-ratio: relation between **s**ignificant **i**nhibitory effects in vitro/ex vivo and the **m**aximal **p**lasma **l**evel after systemic administration in vivo (6.4 ng/ml for SRL). Definition of the SI/DES-ratio: relation between **s**ignificant **i**nhibitory effects in vitro/ex vivo and the drug concentration in **DES **(7.5 mg/ml in the ISAR drug-eluting stent platform). Part I of the study investigated in cytoflow studies the effect of SRL (0.01–1000 ng/ml) on TNF-α induced expression of intercellular adhesion molecule 1 (ICAM-1) in human coronary endothelial cells (HCAEC) and human coronary smooth muscle cells (HCMSMC). Part II of the study analysed the effect of SRL (0.01–1000 ng/ml) on cell migration of HCMSMC. In part III, IV, and V of the study ex vivo angioplasty (9 bar) was carried out in a human organ culture model (HOC-model). SRL (50 ng/ml) was added for a period of 21 days, after 21 and 56 days cell proliferation, apoptosis, and neointimal hyperplasia was studied.

**Results:**

Expression of ICAM-1 was significantly inhibited both in HCAEC (SRL ≥ 0.01 ng/ml) and HCMSMC (SRL ≥ 10 ng/ml). SRL in concentrations ≥ 0.1 ng/ml significantly inhibited migration of HCMSMC. Cell proliferation and neointimal hyperplasia was inhibited at day 21 and day 56, significance (p < 0.01) was achieved for the inhibitory effect on cell proliferation in the media at day 21. The number of apoptotic cells was always below 1%.

**Conclusion:**

SI/MPL-ratio's ≤ 1 (ICAM-1 expression, cell migration) characterize inhibitory effects of SRL that can be theoretically expected both after systemic and local high dose administration, a SI/MPL-ratio of 7.81 (cell proliferation) represents an effect that was achieved with drug concentrations 7.81-times the MPL. SI/DES-ratio's between 10^-6 ^and 10^-8 ^indicate that the described inhibitory effects of SRL have been detected with micro to nano parts of the SRL concentration in the ISAR drug-eluting stent platform. Drug concentrations in DES will be a central issue in the future.

## Background

Sirolimus (SRL, rapamycin) was initially developed as an antibiotic, then as an immunosuppressant, and recently has been identified as one of the most promising novel agents for prevention of human coronary restenosis, both as the compound in drug eluting stents (DES) or after systemic administration. Antimigratory and antiproliferative properties of SRL were first described almost a decade ago by Poon et al. [1] and Dell [2]. In a very short period of time after these initial reports positive effects of SRL were described in animal [3,4] and clinical restenosis studies, using SRL eluting stents [5] or systemic administration [6,7].

Human ex vivo organ culture models have the advantage that the complexity of the restenosis process can be studied in coherent human arterial tissue [8-10]. In the human renal organ culture model [[8], HOC-model] maximal reactive cell proliferation occurs at day 21 after vascular injury and maximal reactive intimal hyperplasia can be detected at day 56. Recently Nührenberg et al. [11] used the HOC-model to demonstrate that SRL in a concentration of 100 ng/ml combines antiproliferative and anti-inflammatory properties.

There is now clear evidence for a contributory role of inflammatory processes to restenosis following vascular injury and stent implantation. Inflammatory cytokines such as tumor necrosis factor-α (TNF-α) upregulate the intercellular adhesion molecule-1 (ICAM-1). Our group has demonstrated in a three-dimensional transfilter co-culture model of leukocyte attack [12] that stimulation of ICAM-1 expression increases adhesion and chemotaxis of human monocytes and triggers reactive proliferation of co-cultured human coronary smooth muscle cells.

Failed systemic restenosis trials in the 80ies and 90ies of the past century have caused an estimated loss of 2.5 billion € to the pharmaceutical industry [13,14], indicating the need for tools to predict the clinical relevance of positive in vitro data. Recently our group has suggested to apply a SI/MPL-ratio, characterizing the relation between a significant antiproliferative effect of a substance in vitro (SI) and the maximal plasma level (MPL) of this substance after systemic administration [13,14]. A SI/MPL-ratio < 1 characterizes an agent that has at least a theoretical chance to achieve positive effects after systemic administration (e.g. positive antiproliferative effects) and after local high dose administration. If the SI/MPL-ratio is > 1, it describes how many times the positive in vitro concentration is above the MPL. In this case the agent has a mere local high dose option, e.g. in a drug eluting stent (DES).

Recently a strong dose depending antiproliferative effect of SRL in human coronary vascular cells has been reported by our group [13]. The antiproliferative effect was achieved with a SI/MPL-ratio's < 1, indicating that the effect might theoretically be achieved both after systemic and local high dose administration [13,14]. Data on the effect of SRL on expression of ICAM-1 and migration in human coronary vascular cells are missing, these issues will be addressed in part I of the current study. In part II of the study we will apply the ex vivo HOC-model to investigate the effect of SRL on reactive cell proliferation, apoptosis, and neointimal hyperplasia. Nührenberg et al. [11] had already demonstrated that SRL in a concentration of 100 ng/mL (more than 10 times the maximal plasma level after systemic administration, MPL) inhibits proliferation and inflammation. In order to approach more closely to the MPL we have investigated the effect of SRL in a concentration of 50 ng/mL.

The current study investigates the effect of SRL on key events of restenosis in a cascade of human in vitro and ex vivo models. A theoretical clinical relevance of the data will be characterized by SI/MPL- [13,14] and, for the first time, by SI/DES-ratio's. The SI/DES-ratio describes the relation between the drug concentration with a significant inhibition in vitro or ex vivo (SI) and the drug concentration in a drug eluting stent (DES)

## Methods

### Cell isolation, cultivation and identification

Endothelial cells from human umbilical veins (HUVEC) were isolated after vaginal delivery by enzymatic disaggregation with collagenase/dispase as described previously [15]. Endothelial cells from human coronary arteries (HCAEC) were purchased at Cambrex Bio-products (Vervier, B). Smooth muscle cells from the human coronary media (HCMSMC) were purchased at Cambrex Bioproducts. Cells were cultured and identified as described [15].

### Sirolimus

Sirolimus (SRL): Rapamycin^®^, Sigma, Deisenhofen, D, 0.01 – 1000 ng/ml, dilution: methanol; MPL: 6.4 ng/ml [6].

### Flow cytometry

For flow cytometry analysis of the expression of ICAM-1 in HCAEC, HUVEC, and HCMSMC cells were trypsinized and seeded into 6-well dishes (5 × 10^4 ^cells). SRL (0.01, 0.1, 1, 10, 100, 1000 ng/ml) was added to the cultures for a period of 18 h. During the last 6 h of SRL incubation, the expression of adhesion molecules was stimulated by adding of TNF-α (20 ng/mL).

After SRL/TNF-α treatment, cells were washed twice with phosphate-buffered saline (pH 7.2) and trypsinized. Cells were resuspended in 100 μL of a FITC-conjugated monoclonal antibody directed against ICAM-1 (clone 84H10, Dianova Immunotech; final concentration 10 μg/mL) and incubated for 20 min at 4°C. A total of 1 × 10^4 ^cells (100% gated) were analyzed immediately with a flowcytometer (BD FACSCalibur, Becton Dickinson, Heidelberg, D).

The effects of SRL (0.01 ng/mL – 1000 ng/mL) on vitality of HCAEC, HUVEC, and HCMSMC was analyzed with propidium iodide using a flowcytometer.

### Boyden chamber

For cell migration studies a Boyden Chamber (QCM™ 24-well colorimetric cell migration assay, Chemicon Europe, UK) was used. HCMSMC were cultured for 48 h in SmBM-culture medium with 1% fetal calf serum (fcs), thereafter a cell supension with 4 × 10^4 ^cells/filter was produced. 300 μl of this suspension was added in serumfree medium to the upper side of the filter, 500 μl supplemented with 10% fcs were added to the inferior side of the filter. The Boyden Chamber was cultured for a period of 24 h, thereafter cells on the upper side of the filters were removed. Filters were stained, dried, and incubated with extraction buffer for 15 min. Finally the optical density was measured at 560 nm.

### The human organ culture model (HOC-Model)

During routine-nephrectomies parts of renal arteries of 15 patients (ages: 66.5 ± 10 years, male/femal: 11/4) were extracted, 15 arteries were suitable for organ culture preparations. In the laboratory, renal artery segments were carefully prepared and sections were made at 3 mm intervals perpendicular to the vessel wall axis [8].

#### Ex vivo ballooning and adding of SRL

Ex vivo angioplasty was carried out as described [8]. After ex vivo ballooning SRL (50 ng/ml) was added to the cultures for a period of 21 days. At each medium exchange the drug was renewed.

#### Cultivation and fixation of organ cultures

Cultivation and fixation of organ cultures was carried out as described earlier [8]. Culture conditions for control groups were exactly the same as described for the angioplasty/SRL group.

#### Analysis of reactive cell proliferation, apoptosis, and neointimal thickening

The effect of SRL (50 ng/mL) on reactive cell proliferation, apoptosis, and neointimal thickening [8] was studied at day 21 and day 56 after ballooning, controls were performed with and without ballooning. Due to the fact that atheroslerosis was present in the vast majority of renal arteries these atherosclerotic areas were defined as "plaque-intima" and had to be differentiated from the neointimal areas forming after angioplasty. The neointima can be differed from the plaqueintima by the very low density of the neointimal structures [8]. Proliferation (PCNA) and apoptosis (TUNEL-technique) of cells was studied in the neointima, plaque-intima, and media.

Proliferation of cells was analyzed with the Proliferating Cell Nuclear Antigen (PCNA). For immunohistological staining paraffin sections were processed using a avidin-biotin immunoperoxidase technique. PCNA-antibodies (Dakopatts Hamburg, D) in a concentration of 1 μg/ml were used as primary antibodies. The number of proliferating cells was calculated as: PCNA positive cells/total cell number × 100.

Apoptotic cells were detected with the TUNEL-technique (in situ cell detection kit, Roche, Mannheim, D) according the manufactures's instructions. The number of apoptotic cells was calculated as: apoptotic cells/total cell number × 100.

### SI/MPL-ratio

The SI/MPL-ratio was calculated in order to describe the relation between a **s**ignificant **i**nhibitory effect in vitro and the **m**aximal **p**lasma **l**evel after systemic administration in vivo [13,14].

### SI/DES-ratio

The SI/DES-ratio was calculated in order to describe the relation between a **s**ignificant **i**nhibitory effect in vitro and the drug concentration in a **DES**.

### Statistical analysis

Data of flow cytometry and migration studies were presented as mean ± S.D. Statistical significance of differences between controls and drug-treated cells was determined by paired Student's t-test. The Mann-Whitney rank-sum test was used to investigate the significance of differences in the human organ culture model. Statistical significance was accepted for *P *< 0.05.

## Results

### Identification of HCAEC, HUVEC, and HCMSMC

Monocultures of HCAEC and HUVEC were identified by positive reaction with antibodies directed against von Willebrand factor and the typical "cobblestone" growth pattern in culture. Monocultures of HCMSMC exhibited the "hill and valley" growth pattern and reacted positively with antibodies against smooth muscle α-actin.

### Effect of SRL on ICAM-1 expression in HCAEC, HUVEC, and HCMSMC

**Figure 1:**
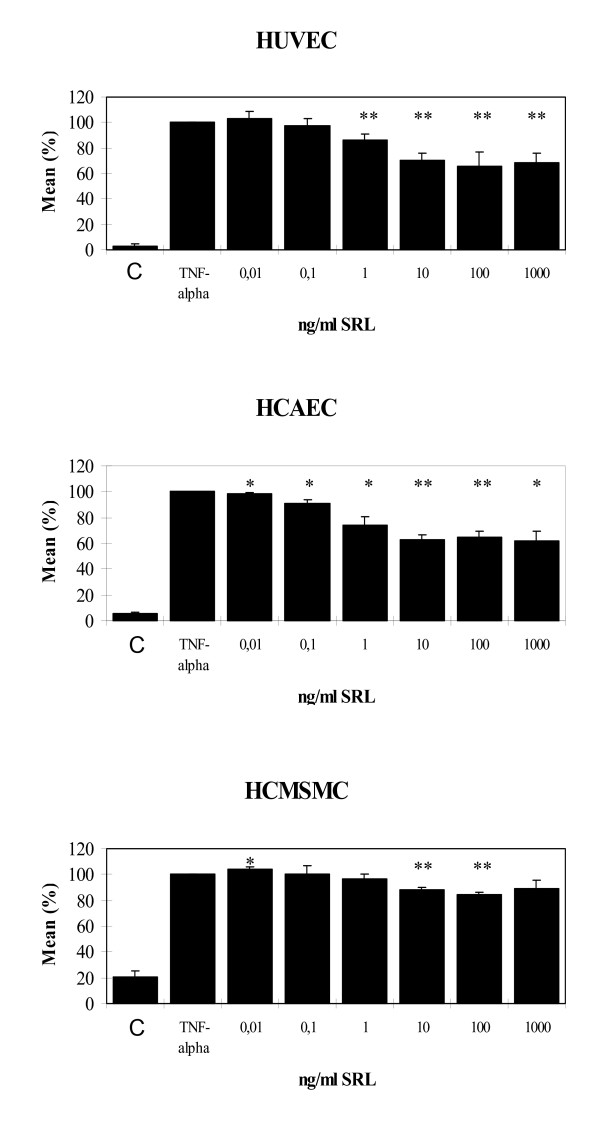
The effects of SRL (0.01, 0.1, 1, 10, 100, 1000 ng/ml) on TNF-α induced expression of ICAM-1 are demonstrated. A dose dependent significant inhibition of ICAM-1 expression was detected in HCAEC (SRL ≥ 0.01 ng/ml), HUVEC (SRL ≥ 1 ng/ml), and HCMSMC (SRL ≥ 10 ng/ml).

The effects of SRL (0.01, 0.1, 1, 10, 100, 1000 ng/ml) on the TNF-α induced expression of ICAM-1 are demonstrated in figure 1. A dose dependent significant inhibition of ICAM-1 expression was detected in HCAEC, HUVEC, and HCMSMC. In comparison to HCMSMC the inhibitory effect of SRL on ICAM-1 expression was pronounced in both HCAEC and HUVEC.

In HCAEC, treatment with TNF-α increased the mean fluorescence levels (%) of ICAM-1 expression 18.83-fold from 5.31% to 100.00%. Incubation of HCAEC with SRL caused a dose dependent inhibition of ICAM-1 expression. After incubation with SRL in concentrations of 0.01, 0.1, and 1 ng/ml expression of ICAM-1 was significantly decreased by 1.13% (p < 0.05), 8.9% (p < 0.05), and 25.73% (p < 0.05), corresponding to SI/MPL-ratio's of 1.563 × 10^-3^, 1.563 × 10^-2^, 0.1563 and SI/DES-ratio's of 1.3 × 10^-9^, 1.3 × 10^-8^, 1.3 × 10^-7^. Incubation of HCAEC with SRL in concentrations of 10, 100, and 1000 ng/mL caused an decrease by 36.92% (p < 0.01), 35.4% (p < 0.01), and 38.4% (p < 0.05), corresponding to SI/MPL-ratio's of 1.563 × 10^-2^, 0.1563, 1.563 and SI/DES-ratio's of 1.3 × 10^-8^, 1.3 × 10^-7^, 1.3 × 10^-6^.

In HUVEC, treatment with TNF-α increased the mean fluorescence levels (%) of ICAM-1 expression 39.53-fold from 2.53% to 100.00%. Incubation of HUVEC with SRL in concentrations of 0.01 ng/ml and 0.1 ng/ml caused a small stimulatory effect of 2.7% (n.s.) and a small inhibitory effect of 2.22% (n.s.). After incubation of HUVEC with SRL in concentrations of 1, 10, 100, and 1000 ng/ml expression of ICAM-1 was inhibited by 13.76% (p = 0.01), 29.85% (p < 0.01), 34.08% (p < 0.01), and 31.8% (p < 0.01), corresponding to SI/MPL-ratio's of 0.1563, 0.01563, 0.1563, 1.56 and SI/DES-ratio's of 1.3 × 10^-7^, 1.3 × 10^-8^, 1.3 × 10^-7^, 1.3 × 10^-6^.

In HCMSMC, treatment with TNF-α increased the mean fluorescence levels (%) of ICAM-1 expression 4.93-fold from 20.29% to 100.00%. Incubation of HCMSMC with SRL in concentrations of 0.01, 0.1, and 1 ng/ml caused a small stimulatory effects of 4.37% (p < 0.05; SI/MPL-ratio: 1.563 × 10^-3^; SI/DES-ratio: 1.3 × 10^-9^) and 0.43% (n.s.) and a small inhibitory effect of 3.35% (n.s.). After incubation of HCMSMC with SRL in concentrations of 10, 100, and 1000 ng/ml expression of ICAM-1 was inhibited by 11.75% (p < 0.01; SI/MPL-ratio: 0.01563; SI/DES-ratio: 1.3 × 10^-8^), 15.92% (p < 0.01; SI/MPL-ratio: 1.563 × 10^-2^; SI/DES-ratio: 1.3 × 10^-8^), and 11.15% (n.s.).

### Effect of SRL on migration of HCMSMC

**Figure 2:**
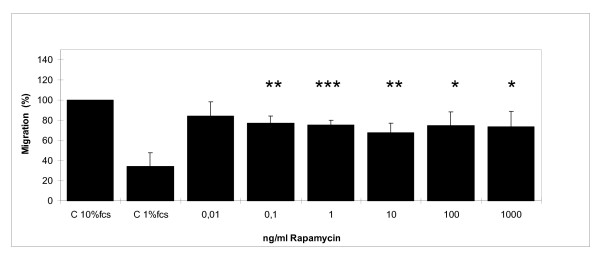
The effects of SRL (0.01, 0.1, 1, 10, 100, 1000 ng/ml) on migration of HCMSMC are demonstrated. A significant inhibition of cell migration was detected after adding of SRL in concentrations ≥ 0.1 ng/ml (* = p < 0.5; ** = p < 0.01; *** = p < 0.001; SI/MPL-ratio ≥ 0.016; SI/DES-ratio ≥ 1.3 × 10^-8^).

The effects of SRL (0.01, 0.1, 1, 10, 100, 1000 ng/ml) on migration of HCMSMC are demonstrated in figure 2. A significant inhibition of cell migration was detected after adding of SRL in concentrations ≥ 0.1 ng/ml (SI/MPL-ratio ≥ 0.016; SI/DES-ratio ≥ 1.3 × 10^-8^).

After incubation of HCMSMC with SRL in concentrations of 0.01, 0.1, and 1 ng/ml migration of HCMSMC was inhibited by 15.74% (n.s.), 23.1% (p < 0.01), and 24.67% (p < 0.001), corresponding to SI/MPL-ratio's of 1.563 × 10^-2^, 0.1563 and SI/DES-ratio's of 1.3 × 10^-8^, 1.3 × 10^-7^. Incubation of HCMSMC with SRL in concentrations of 10, 100, and 1000 ng/mL caused an decrease of cell migration of 32.09% (p < 0.01), 25.28% (p < 0.05), and 26.34% (p < 0.05), corresponding to SI/MPL-ratio's of 1.563 × 10^-2^, 0.1563, 1.563 and SI/DES-ratio's of 1.3 × 10^-8^, 1.3 × 10^-7^, 1.3 × 10^-7^.

### Effect of SRL on proliferation, apoptosis, and neointimal hyperplasia

**Figure 3:**
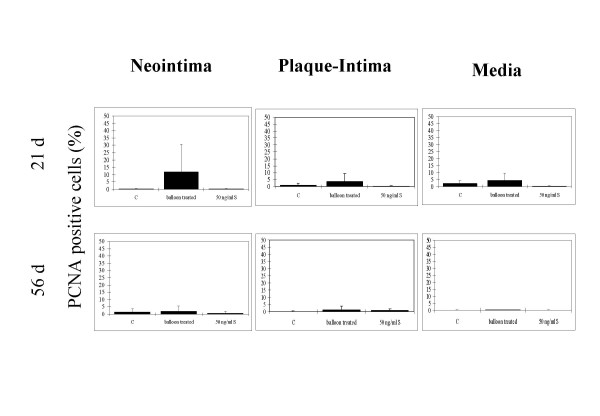
In the ex vivo HOC-model the effect of a 21 days incubation with SRL (50 ng/ml) on reactive cell proliferation was studied in the neointima, plaque-intima, and media at day 21 and day 56. SRL inhibited reactive cell proliferation, statistical significance was achieved for the inhibitory effect on cell proliferation in the media at day 21 (SI/MPL-ratio: 7.81).

**Figure 4:**
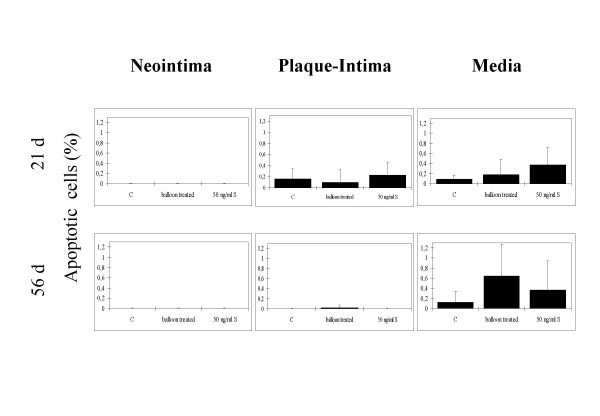
The effect of incubation of the ex vivo HOC-model with SRL (50 ng/ml) on apoptosis was studied in the neointima, plaque-intima, and media at day 21 and day 56.

**Figure 5:**
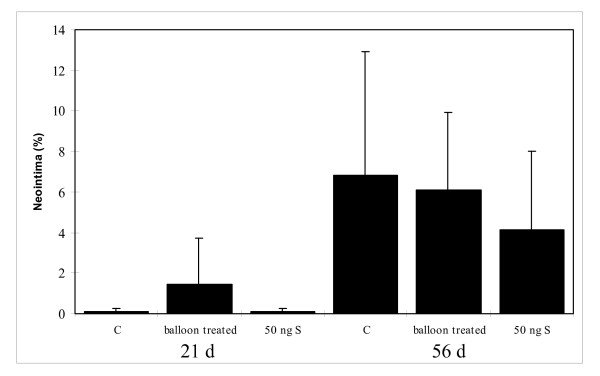
In the ex vivo HOC-model the effect of 21 days incubation with SRL (50 ng/ml) on neointimal hyperplasia was studied 21 days and 56 days after ballooning. SRL inhibited neointimal hyperplasia, statistical significance was not achieved.

In the current human ex vivo study the effect of 21 days incubation with SRL (50 ng/ml) on reactive cell proliferation (Fig. 3), apoptosis (Fig. 4), and neointimal hyperplasia (Fig. 5) was studied 21 days and 56 days after ballooning. SRL inhibited both reactive cell proliferation and reactive neointimal hyperplasia, statistical significance was achieved merely for the inhibitory effect on cell proliferation in the media at day 21. The number of apoptotic cells was always very low and did not increase 1%.

21 days after angioplasty (n = 6) cell proliferation in the neointima, plaque-intima, and the media was increased by 11.89% (n.s.), 3.73% (n.s.), and 4.27% (n.s.) in comparison to cell proliferation before angioplasty. After incubation with SRL (50 ng/ml) for a period of 21 days no cell proliferation was detected in the neointima, cell proliferation in the plaque-intima and media was decreased by 93.3% (n.s.) and 95.78% (p < 0.01; SI/MPL-ratio: 7.81). 56 days after angioplasty (n = 10) cell proliferation in the human organ cultures was very low and did not increase 2%. In the neointima, plaque-intima, and media cell proliferation was increased by 1.96% (n.s.), 1.25% (n.s.), and 0.23% (n.s.) in comparison to cell proliferation before angioplasty and decreased by 70.41% (n.s.), 32% (n.s.), and 52.17% (n.s.) after SRL incubation.

The number of apoptotic cells in the artery segments was always very low and did not increase 1%. Apoptotic cells was detected merely in the media, almost no apoptosis was found in the neointima and plaque-intima. 21 days after angioplasty (n = 6) apoptosis in the media was increased by 0.18% (n.s.) in comparison to the percentage of apoptotic cells before angioplasty, 56 days after angioplasty by 0.64% (n.s.). Incubation with SRL caused both stimulating and inhibiting effects. At day 21 the number of apoptotic cells was increased by 52.6% in comparison to the angioplasty group (n.s.), at day 56 inhibited by 42.19% (n.s.).

Neointimal hyperplasia after angioplasty was increased by 1.44% at day 21 (n = 5) and 6.11% at day 56 (n = 10) in comparison to neointimal hyperplasia before angioplasty. After a 21-days incubation with SRL (50 ng/ml) no neointimal hyperplasia was detected at day 21 and a reduction by 31.26% was detected at day 56 (n.s.).

## Discussion

The current study reports on the effects of SRL in various human in vitro/ex vivo models and evaluates the theoretical clinical relevance of the data by SI/MPL- and SI/DES-ratio's.

Expression of ICAM-1 is one of the crucial events for adhesion and chemotaxis of human monocytes from the circulating blood into the coronary artery wall. In the current study TNF-α induced expression of ICAM-1 was significantly inhibited by SRL both in endothelial and smooth muscle cells. The corresponding SI/MPL-ratio's of 0.00156 (HCAEC), 1.56 (HCMSMC), and 0.156 (HUVEC) indicate that the inhibitory effects may be expected theoretically both after systemic and local high dose administration. In contrast to the current data Minhajuddin et al. [16] reported that SRL in a concentration of 5 ng/ml inhibits mammalian target of rapamycin (mTOR) but stimulates thrombin-induced expression of ICAM-1 in endothelial cells. In the current study SRL concentrations of 1 ng/ml and 10 ng/ml inhibited expression of ICAM-1 both in endothelial and smooth muscle cells. Surprisingly, a small stimulatory effect on ICAM-1 expression was detected in HCMSMC after incubation with SRL in a concentration of 0.01 ng/ml. The contrasting data may be partially explained by the fact that Minhajuddin et al. [16] reported on the effect of thrombin-induced expression of ICAM-1, whereas the current study described the effect of TNF-α-induced ICAM-1 expression. Moreover activation of ICAM-1 expression via NF-κB is complicated by redundant pathways and differences exist in endothelial cells and smooth muscle cells, as reported earlier by our group [15]. Recently, a role of c-Jun N-terminal kinase (JNK) in thrombin-induced ICAM-1 expression in endothelial cells was described, a mechanism that is dependent on Gα, Gβγ, Rasw, Rac1, and the Src kinase family [17].

In the current study migration of HCMSMC was inhibited after administration of SRL in concentrations ≥ 0.1 ng/ml. The corresponding SI/MPL-ratio of 0.016 indicates that the antimigratory effect was achieved with 1.6% of the maximal plasma level after oral application, resulting in both a systemic and local high dose option for this effect. These data are in accordance with data in the literature [1,18]. Poon et al. [1] reported that pretreatment with SRL in a concentration of 2 ng/mL inhibited PDGF-induced migration of rat aortic smooth muscel cells in a modified Boyden chamber. Sakakibara et al. [18] reported that SRL in a concentration of 10 nM (corresponding to a concentration of 9.14 ng/ml) markedly diminuished fibronectin induced migration of a rat aortic smooth muscle cells and of a human arterial smooth muscle cell line (E47). According to the authors the fibronectin induced migration of rat and human smooth muscle cells may be inhibited through a pathway that involves at least alpha(v)beta[3]-integrin, phosphatidylinositol 3-kinase (PI3K), the mammilian target of rapamycin (mTOR), and S6 kinase (S6K). Migration in the current study was carried out in a Boyden chamber against culture medium with 10% fetal calf serum, therefore it's not clear whether the medium contained fibronectin or not. The missing dose dependency in the current study is difficult to explain. Poon et al. [1] and Sakakibara et al. [18] described an inhibitory effect of SRL on PDGF-induced [1] respectively fibronectin-induced [18] migration of rat aortic smooth muscle cells. Both groups did not investigate the effect of various SRL concentrations. In contrast to the current data Parry et al. [19] reported that low concentrations of sirolimus inhibited merely cell proliferation and not cell migration.

In the ex vivo part of the study the effect of SRL in a concentration of 50 ng/ml on cell proliferation, apoptosis, and neointimal hyperplasia was studied. An antiproliferative effect of SRL on cell proliferation was first described by Dell [2]. Marx et al. [20] reported of a significant inhibitory effect of SRL in a concentration of 1 ng/ml in SMC from rat and human aorta (SI/MPL-ratio: 0.16). These data were confirmed later by data of our group with human coronary smooth muscle cells [[13]; SI/MPL-ratio: 0.16]. Recently Nührenberg et al. [11] reported of a significant antiproliferative effect of SRL in a concentration of 100 ng/ml in the HOC-model at day 21 after angioplasty (SI/MPL-ratio: 15.63). In accordance with these reports the current study describes a significant antiproliferative effect of SRL in a concentration of 50 ng/ml in the HOC-model at day 21 after angioplasty (SI/MPL-ratio: 7.81). Both the ex vivo study of Nührenberg et al. [11] and the current ex vivo data were carried out with drug concentrations 15.63-times respectively 7.81-times the MPL. Therefore these results merely prove the antiproliferative effect of SRL in DES, not after systemic administration as the prior in vitro studies of Marx [20] and our group [14].

Neointimal hyperplasia was inhibited after incubation with SRL in a concentration of 50 ng/ml at day 21 and day 56, statistical significance was not achieved. These data are in accordance with many reports in rabbit [21] and porcine [3,4] experimental models. Experimental respectively ex vivo data of intimal hyperplasia correspond to a certain degree to clinical angiographic restenosis data. However due to the fact that the current study was carried out with a concentration of SRL 7.81-times the MPL after oral administration of SRL, it can merely be compared with clinical studies with SRL-eluting stents [5] and not with studies describing the effect of oral application [6,7].

Apoptotic effects did not play a major role in the restenosis process of the HOC-model, the number of apoptotic cells never increased 1%. In contrast to the current data apoptotic cell death has been documented in numerous animal models of vascular injury [22-24]. Pollman et al [23] reported that the rapid wave of apoptosis resulting from vascular injury appears to involve a redox sensitive pathway, because local administration of antioxidants has minimized cell loss. Normocholesterolemic and hypercholesterolemic rabbits display similar profile of early postinjury apoptosis, but hypercholesterolemia enhances apoptosis in the neointima at two weeks after injury [23]. This observation has led to the hypothesis that macrophages present in the vascular lesions of the hypercholesteremic rabbits may contribute to SMC turnover. Macrophage involvement has also been implicated in apoptosis observed after stent implantation in rabbit vessels [25].

In the literature the amount of SRL in DES is described as μg/stent or μg/mm^2^, informations that are useless for a correlation with in vitro/ex vivo drug concentrations. To our best knowledge the only exception is a report of Wessely et al. [26], describing SRL concentrations of 7.5 mg/ml in the ISAR drug eluting stent platform. The relation between the significant inhibitory effects in the current study and the drug concentrations in the ISAR drug-eluting stent platform results in SI/DES-ratio's of 10^-6 ^and 10^-8^.

## Limitations of the study

The adhesion molecule ICAM-1 is merely one of a large number of inflammation markers. In the current study we focused on expression of ICAM-1 because our laboratory has already characterized precisely the TNF-α induced stimulation of ICAM-1 expression in HCAEC, HUVEC, and HCMSMC [27].

Although ex vivo models are very attractive they are hampered by high standard deviations due to the preparation procedure [8]. The arteries are cut at the frontal sides and the adventitial side. Balloon injury is merely one additional stimulus in this activated situation. If the ex vivo HOC-model is used to study the effect of agents on key events of restenosis, significant differences to untreated controls cannot be achieved.

SI/MPL- and SI/DES-ratio's are merely a theoretical help to evaluate the clinical relevance of in vitro/ex vivo data.

## Conclusion

SI/MPL-ratio's ≤ 1 (ICAM-1 expression, cell migration) characterize inhibitory effects of SRL that can be theoretically expected both after systemic and local high dose administration, a SI/MPL-ratio of 7.81 (cell proliferation) represents an effect that was achieved with drug concentrations 7.81-times the MPL. SI/DES-ratio's between 10^-6 ^and 10^-8 ^indicate that the described inhibitory effects of SRL have been detected with micro to nano parts of the SRL concentration in the ISAR drug-eluting stent platform. It remains to be elucidated whether SRL concentrations in DES can be drastically reduced without loosing the convincing anti-restenotic effects in vivo.

Due to the fact that the application of the SI/MPL-ratio [13,14] might have stopped many negative systemic restenosis trials in the 80ies and 90ies, saving an estimated loss of 2.5 billion € of the pharmaceutical industry [14], the usefulness of the SI/MPL-ratio is convincing. Due to antiproliferative SI/MPL-ratio's > 1, prednisolone (SI/MPL-ratio: 7.1; 28), dexamethasone (SI/MPL-ratio: 6.6; 28), cyclosporine A (SI/MPL-ratio: 11.1; 29), iloprost (SI/MPL-ratio: 10; 30), triazolopyrimidine (SI/MPL-ratio: 4.86; 31), diltiazem (SI/MPL-ratio: 104.2; 32), and abciximab (SI/MPL-ratio: 11.4; 33) should not have been applied in clinical studies rationally based on a significant antiproliferative effect. Antiproliferative SI/MPL-ratio's < 1 have been reported of the cytostatic agents cytarabine (SI/MPL-ratio: 0.1; 34), doxorubicine (SI/MPL-ratio: 0.83; 34), etoposide (SI/MPL-ratio: 0.0021; 34), paclitaxel (SI/MPL-ratio: 0.002; 30) and of the immunosuppressive agents hydrocortisone (SI/MPL-ratio: 0.043; 28), sirolimus (SI/MPL-ratio: 0.01; 13), and mycophenolate mofetil (SI/MPL-ratio: 0.0011; 13). These agents have at least a theoretical chance to reproduce an antiproliferative effect after systemic administration.

The application of a SI/DES-ratio is suggested for the first time in the current study. Drug concentrations in current DES are extremely high [26], resulting in SI/DES ratio's between 10^-6 ^and 10^-8^. This is overdone and may partially cause the problems of late thrombosis in DES [35]. Probably antiproliferative SI/DES-ratio's between 10^-1 ^to 10^-2 ^are sufficient to create a significant local antiproliferative effect.

Drug concentrations in DES will be the central issue of the future. It has to be demonstrated, if the SI/DES-ratio can close a little bit the gap between bench and bedside by keeping the focus on the relation between effective drug concentrations in vitro and the local concentration in DES.

## Competing interests

The author(s) declare that they have no competing interests.

## Authors' contributions

All authors read and approved the final manuscript. RV, RB, VH, JK designed the study, RV wrote the manuscript. SZ and RB carried out PCNA and apoptosis studies, FM and LvM have done cytoflow studies. Cell migration studies were done by RB, FF and RB carried out morphological studies, JB, JG, and MK supplied renal artery segments and critically discussed the manuscript.

## Pre-publication history

The pre-publication history for this paper can be accessed here:
